# Twin-lattice atom interferometry

**DOI:** 10.1038/s41467-021-22823-8

**Published:** 2021-05-05

**Authors:** Martina Gebbe, Jan-Niclas Siemß, Matthias Gersemann, Hauke Müntinga, Sven Herrmann, Claus Lämmerzahl, Holger Ahlers, Naceur Gaaloul, Christian Schubert, Klemens Hammerer, Sven Abend, Ernst M. Rasel

**Affiliations:** 1 Zentrum für angewandte Raumfahrttechnologie und Mikrogravitation (ZARM), Universität Bremen, Bremen, Germany; 2 Institut für Quantenoptik, Leibniz Universität Hannover, Hannover, Germany; 3 Institut für Theoretische Physik, Leibniz Universität Hannover, Hannover, Germany; 4 German Aerospace Center (DLR), Institute for Satellite Geodesy and Inertial Sensing, Bremen, Germany; 5 German Aerospace Center (DLR), Institute for Satellite Geodesy and Inertial Sensing, Hannover, Germany

**Keywords:** Ultracold gases, Matter waves and particle beams, Quantum metrology

## Abstract

Inertial sensors based on cold atoms have great potential for navigation, geodesy, or fundamental physics. Similar to the Sagnac effect, their sensitivity increases with the space-time area enclosed by the interferometer. Here, we introduce twin-lattice atom interferometry exploiting Bose-Einstein condensates of rubidium-87. Our method provides symmetric momentum transfer and large areas offering a perspective for future palm-sized sensor heads with sensitivities on par with present meter-scale Sagnac devices. Our theoretical model of the impact of beam splitters on the spatial coherence is highly instrumental for designing future sensors.

## Abstract (web summary)

Atom interferometers can be useful for precision measurement of fundamental constants and sensors of different type. Here the authors demonstrate a compact twin-lattice atom interferometry exploiting Bose-Einstein condensates (BECs) of 87 Rb atoms.

## Introduction

Atom interferometers measure inertial forces^1–3^, atomic properties, and quantities like the photon recoil^4^ or the gravitational constant^5^ with high precision and accuracy. Modern fields of application comprise navigation and observation of Earth’s gravity and rotation, as well as terrestrial and space-borne gravitational-wave detection in the infrasound domain^6–11^. Achieving interferometers with the needed space-time areas on the order of one m·s remains challenging, though.

The sensitivity of atom interferometers towards inertial forces^12^ and gravitational waves increases linearly with the differential kinetic momentum, and, hence, the latter is exploited as a lever. Benchmark experiments have so far realized asymmetric and symmetric momentum transfer with Raman diffraction^13–15^ and sequential and higher-order Bragg transitions^16–19^, as well as Bloch oscillations (BOs)^20–22^. Other experiments, where both interferometer arms were equally accelerated and, thus, the relative momentum in the interferometer remained unaffected, involve even higher numbers in photon transfer^4,23^.

Large momentum transfer is especially of interest for increasing the sensitivity of Sagnac interferometers, which scales with the enclosed area. Compared to laser gyroscopes exhibiting resolutions of up to $1{0}^{-11}\,{\rm{rad}}\,{({\rm{s}}\sqrt{{\rm{Hz}}})}^{-1}$^24^, in matter-wave interferometers^1,14,25–28^ smaller areas suffice to reach high sensitivities. However, forming the required loop size is still challenging and drives the dimensions of the apparatus.

Here, we present twin-lattice interferometry as a method to form symmetric interferometers featuring matter waves with large relative momentum. By employing two counterpropagating optical lattices^29^, we create from a Bose–Einstein condensate (BEC) a Mach–Zehnder-type interferometer, made of wave packets with a differential momentum of >400 photon recoils, the largest reported so far. In this way, we realize a Sagnac loop with an area of *A* = 7.6 mm^2^ on a baseline of only 2.43 mm. Compared to previous approaches twin-lattice interferometry with BECs displays several advantages: (i) It enables large and symmetric momentum transfer, and, hence, large space-time areas in a simple and efficient way by combining double Bragg diffraction (DBD)^18,30^ with symmetric BOs^31^. (ii) The symmetry of our geometry will suppress systematic effects such as diffraction phases, a common challenge in current implementations^4,19,32^. (iii) Combined with a delta-kick-collimated BEC source, we achieve high scalability marked by low atom and contrast loss. According to our theoretical model, the interference contrast achieved with this method so far is solely limited by technical features of our device. Consequently, twin-lattice interferometry provides the perspective to create large Sagnac loops in a swift manner in compact devices outperforming today’s approaches^1,28,33^. Furthermore, the method brings within reach the momentum transfer of thousands of photon recoils and opens up exciting perspectives for many applications, in particular gravitational-wave detectors^6,8,10,11^.

## Results

### Experimental setup

**Fig. 1:**
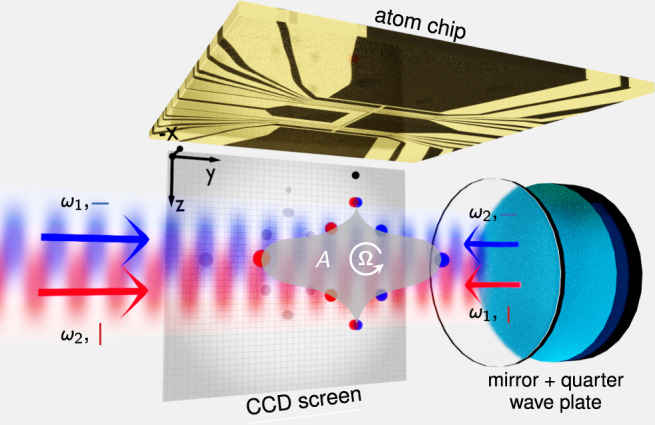
Twin-lattice setup. The twin lattice is formed by retroreflecting light featuring two frequencies with linear orthogonal polarization. A quarter-wave plate in front of the retroreflector alters the polarization to generate two counterpropagating lattices (indicated in red and blue). After release from the atom chip and state preparation, the BEC is symmetrically split and recombined by the lattices driving double Bragg diffraction (DBD) and Bloch oscillations (BOs). In this way, the interferometer arms form a Sagnac loop enclosing an area *A* (shaded in gray) for detecting rotations **Ω**. The interferometer output ports are detected on a CCD chip by absorption imaging.

The setup for our twin-lattice interferometer is shown in Fig. 1. The twin lattice is formed by retroreflecting a laser beam featuring two frequencies with linear orthogonal polarization through a quarter-wave plate (Supplementary section 1). The beam of 3.75 mm waist and up to 1.2 W power travels below and parallel to the horizontally aligned surface of an atom chip.

The latter serves to generate BECs of up to 1.5 × 10^4^ ^87^Rb atoms in the magnetic state *F* = 2, *m*_*F*_ = 2 in a Ioffe–Pritchard trap with frequencies of (43, 344, 343) Hz as detailed in ref. ^34^. The BECs are released from the trap into free fall by switching off the magnetic field and are, after a free expansion of 5.4 ms, again exposed to the same field for 0.3 ms. In this way, they experience a delta kick, which acts as a collimating lens and results in a narrow momentum distribution^35^, that is, a residual expansion rate along the lattice of *σ*_*v*_ = 0.18 mm s^−1^ or 0.03ℏ*k*, corresponding to an effective 1D temperature of 340 pK. The collimation is crucial for efficient manipulation of the BECs via DBD in combination with momentum transfer through BOs^36^. Immediately after the delta kick, an adiabatic rapid passage of 9 ms duration transfers 90–95% of the BEC to the non-magnetic state *F* = 2, *m*_*F*_ = 0 by coupling the Zeeman levels with a chirped radio-frequency pulse. This state is separated from the others by temporarily applying a vertical magnetic field gradient. The effect of such a Stern–Gerlach-type experiment on the atomic expansion rate is negligible. Atoms are detected by absorption imaging with a CCD camera at a maximum observable free-fall time of 35.5 ms.

### Interferometer sequence

**Fig. 2:**
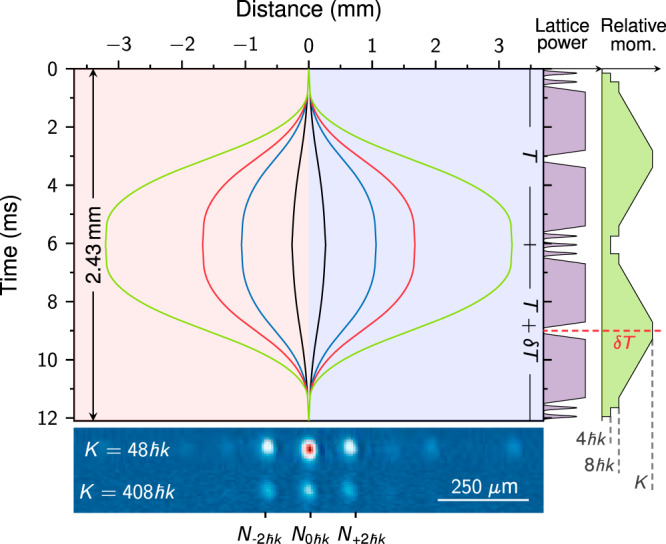
Twin-lattice scheme and sequence. Space-time trajectories of the wave packets during the interferometer depicted for momentum transfers of *K* = (24, 128, 208, 408)ℏ*k* (black, blue, red, green) with distances given relative to the release point. Below: absorption images of the interferometer output ports for *K* = 48ℏ*k* and 408ℏ*k* after 35.5 ms of free fall. Right: temporal sequence of the twin-lattice laser power and the relative momentum *K* in the interferometer. DBD is driven by Gaussian-shaped pulses of 37.5 μs width. BOs for acceleration and deceleration are realized via a linear frequency ramp of 2 ms with 200 μs of loading and unloading time. For contrast analysis, the free-evolution time in the second half is modified by *δT* with respect to the first half.

The interferometer is generated by a sequence of DBD processes and BOs in the twin lattice. Figure 2 shows the resulting space-time trajectories of the wave packets exemplary for momentum transfers of *K* = (24, 128, 208, and 408) ℏ*k* together with the corresponding temporal sequence of lattice power and change in relative momentum.

First, the twin lattice is exploited to induce two successive first-order DBD processes^18^ so that the BEC is split into two wave packets separating with a mean momentum of ±4ℏ*k*, respectively. The light pulses driving DBD have a Gaussian-shaped temporal envelope of 37.5 μs width. Owing to the use of delta-kick-collimated BECs, we achieve a transfer efficiency of 98.8% per recoil in these processes. Hereafter, the BECs are loaded into the counterpropagating lattices. During this process, the lattice intensity is linearly increased within 200 μs and the velocity adjusted to the comoving BECs^37^. Each wave packet is accelerated by its copropagating lattice via BOs during 2 ms and gains additional momentum of up to 200ℏ*k*. For the release from the lattices, the intensity is lowered again linearly. In this way, we obtain an efficiency of up to 99.93% per recoil for BOs in our interferometers. In addition, we have realized a single beam splitter, where the wave packets have been accelerated up to a differential momentum of *K* = 1008ℏ*k* with a Bloch efficiency of 99.87% per recoil. Presently, the transferred number of photon recoils is limited solely by the free-fall time and laser power available in our apparatus.

In the interferometer, the accelerated wave packets evolve freely for 200 μs, before their motion is first slowed down via BOs to ±4ℏ*k*, then inverted via successive DBD and again accelerated via BOs. After a second free evolution of 200 μs, the velocities of the wave packets are reduced to ±4ℏ*k* to recombine them via the last DBD process resulting in three output ports with mean momenta of ±2ℏ*k* and 0ℏ*k* showing interferences. In total, the free evolution of the wave packets and their interaction with the light pulses amount to a duration of 2*T* = 12.1 ms.

The signal of our twin-lattice interferometer is defined as the normalized number of atoms detected in the output ports *p* = (*N*_+2ℏ*k*_ + *N*_−2ℏ*k*_)/(*N*_+2ℏ*k*_ + *N*_−2ℏ*k*_ + *N*_0ℏ*k*_), where *N*_±2ℏ*k*_ are the atom numbers with ±2ℏ*k* and *N*_0ℏ*k*_ with 0ℏ*k* momentum. The absorption images at the bottom of Fig. 2 show the output ports for two experimental realizations where the wave packets in the interferometer have had a momentum splitting of *K* = 48ℏ*k* or 408ℏ*k*, respectively.

### Contrast analysis

**Fig. 3:**
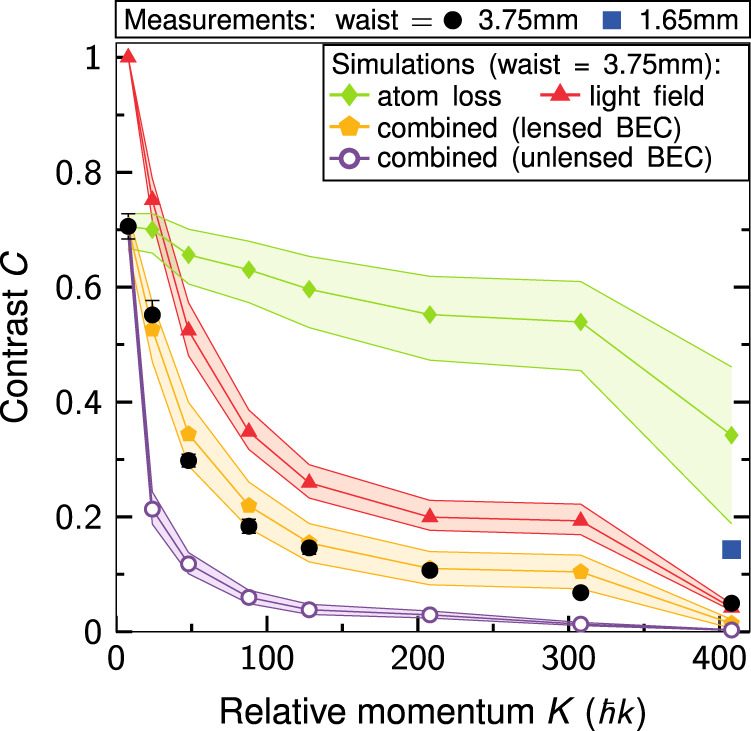
Contrast analysis. Experimental (black circles) and theoretical contrast *C* of our twin-lattice interferometer in dependence of relative momentum *K* for a laser beam waist of 3.75 mm. We assume two effects dominantly contributing to the observed loss of contrast: The first is atom loss due to nonadiabatic transitions (green diamonds). The second effect is a local inhomogeneous dipole force due to light field distortions (red triangles). Combining the simulation of both effects (orange pentagons) leads to a reasonable agreement with the experimental observations. According to our model, a non-collimated BEC (open violet circles) would suffer more strongly from light field distortions and exhibit a significantly lower contrast due to its larger cloud size. Since we assumed equal atom losses for DBD and BOs as for the collimated cloud, this presents an upper bound for the contrast. The shaded areas represent confidence intervals of the simulation, determined by atom number and lattice depth uncertainty. The error of the experimental data is obtained by the standard error of the Gaussian fit in Fig. 6 and lies below the marker size for most of the data points. The blue square shows a measurement, where the contrast has been improved by reducing the twin-lattice beam waist, and, hence, spatial distortions of the twin-lattice beam due to diffraction at the atom chip.

We evaluate the contrast of our interferometer statistically as described in detail in “Methods.” As illustrated in Fig. 3, the measured interference contrast *C* (black dots) clearly decreases with an increasing number of transferred momenta. Confirmed by simulations, we attribute the contrast loss to two main effects.

The first effect stems from atom losses arising during DBD and BOs. On the one hand, atoms that have not been Bragg diffracted to the desired momentum states, and therefore have not performed BOs, still give rise to an offset signal in the number of detected atoms. On the other hand, losses during BOs remove atoms from the interferometer and reduce the number of coherent atoms. To increase the relative momentum *K*, we apply larger accelerations during BOs causing increased Landau–Zener losses. Both loss mechanisms lead to a reduced contrast of about *C* = 0.35 for *K* = 408ℏ*k* (green diamonds, Supplementary sections 2 and 3).

The second cause for the contrast reduction is dephasing due to an imperfect Gaussian beam profile (Supplementary Fig. 1 and section 4). Such perturbations result, for example, from light diffraction on an edge of the atom chip and induce spatially variable dipole forces along the two interferometer arms. Different atomic trajectories according to the ensemble’s spatial distribution in combination with path-dependent dipole forces lead to a spatially varying phase across the wave packets^38–40^ and give rise to a loss of contrast (red triangles). Combining the models for atom loss and dephasing processes (orange pentagons) enables us to match the observed contrast. We base our analysis of the beam profile on a model assuming a mere clipping of the twin-lattice beams at one edge of the chip. Hereby, the relative magnitude of the intensity perturbations represents the only free fitting parameter. The results in Fig. 3 have been obtained with a value equal to 9% of the twin-lattice depth, which is compatible with the measured beam profile. The effect of wavefront errors on the contrast loss is expected to be smaller by a factor of ten^40^.

In our simulation, the loss of contrast critically depends on the wave packet size (Supplementary Fig. 2). The twin-lattice interferometer, therefore, benefits from the small spatial extent of our delta-kick-collimated BEC, exhibiting Thomas–Fermi radii of *R* = (32, 39, 30) μm at the end of the interferometer. In the absence of delta-kick collimation, these radii increase by a factor of three in *y*- and *z*-direction. Since a larger ensemble samples over more light field distortions and, hence, suffers from stronger dephasing, it leads to a significant contrast reduction (open violet circles). Our calculation even overestimates the contrast of the non-collimated BEC, since it is performed assuming the same efficiencies for DBD and BOs as in the case of delta-kick-collimated BECs.

To experimentally verify the impact of the spatial profile of the light field on the interferometric contrast, we have reduced the beam diameter by roughly a factor of two compared to our original setup. The smaller diameter lowers light field distortions caused by diffraction at the atom chip and other apertures. In this way, we are able to triple the measured contrast in our largest interferometer (*K* = 408ℏ*k*) to *C* = 0.14 (blue square). In our simulations, a reduction of the beam diameter leads to an almost unperturbed beam and, therefore, to a significantly improved contrast, mainly determined by atom loss (green line in Fig. 3). The remaining discrepancy can be explained by the fact that the measured beam profile (*w* = 1.65 mm) still features distortions in the order of 5% due to multiple diffractions.

## Discussion

We have employed twin-lattice interferometry for large symmetric momentum transfer and observed spatial coherence up to a maximum splitting of *K* = 408ℏ*k*, the largest momentum separation in an interferometer reported so far. In comparison to previous results, our method is not as strongly limited by atom loss as the benchmark experiment in ref. ^16^. Moreover, diffraction phases should be greatly suppressed as in ref. ^29^; however, the inertial noise in our current setup prevents us from confirming this experimentally. Given the use of two instead of three light fields, our geometry is even more symmetric than^29^. Indeed, for ideal mode overlap, our twin-lattice interferometer should be by construction not susceptible to light shifts that were reported in ref. ^21^.

Our symmetric geometry also relaxes laser power requirements compared to an asymmetric scheme. For the same momentum transfer, the acceleration of only a single interferometer arm would require larger lattice depths causing not only higher atom losses due to spontaneous scattering but also an even lower contrast due to the light field distortions.

Compared to former symmetric schemes, we significantly exceed the contrast observed in the double Raman interferometer^13^ and our own double Bragg interferometer^18^. Our experimental studies, as well as theoretical simulations, reveal that only technical reasons, in particular spatial distortions of the twin lattice, limit the current beam splitter efficiency and generally the scaling of our method.

In fact, our theoretical model for Gaussian-shaped beams and experimental results show that in the ideal case of an undisturbed lattice, neither the Landau–Zener losses nor the differential dipole force arising due to the Gaussian waist will be critical. For a twin lattice featuring spatial intensity fluctuations in the order of 0.5% of *V*_0_, the model predicts a contrast of >90% at *K* = 408ℏ*k*. An additional absolute Stark shift compensation may help to reduce the effect of residual local inhomogeneities and maintain a high contrast by adding a light field of opposite detuning^4,17^.

Twin-lattice interferometry allows to efficiently create large Sagnac areas in comparably short time and space. To evaluate the level of miniaturization and to compare the geometry obtained with our method to other sensors, we introduce a compactness factor (*L*·*τ*)^−1^, which is the inverse product of interferometer time *τ* and baseline *L*.

**Fig. 4:**
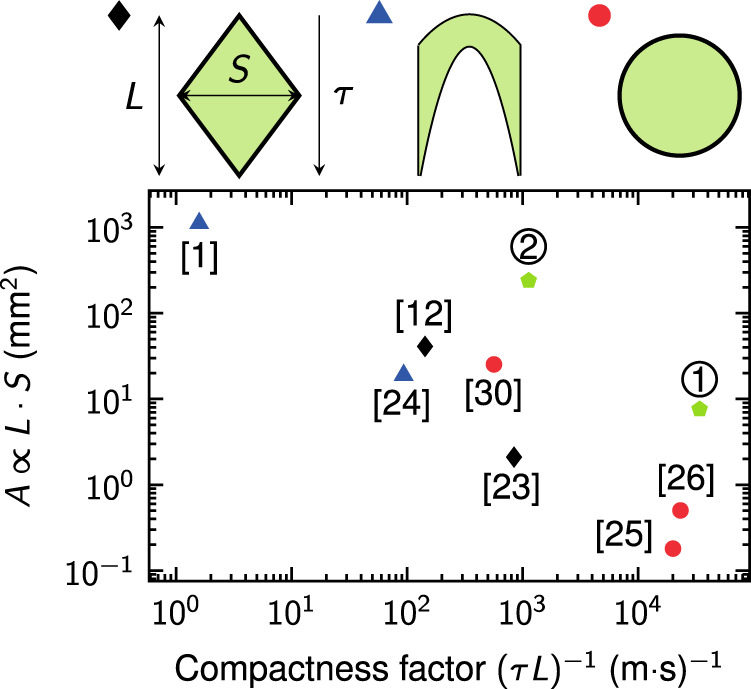
Sensor comparison. Comparison of the effective area *A* enclosed by the interferometer arms as a function of the compactness factor (*τ* · *L*)^−1^, the inverse of the product of interferometer duration *τ* and baseline *L*. In principle, the area increases linearly with the maximum separation *S* and the baseline *L*. For high compactness and large Sagnac areas, it is advantageous to reach large separations *S* in short times *τ* at a balanced baseline *L*. The presented experiments employ either Mach–Zehnder-type topologies (black diamonds)^14,25^, butterfly geometries (blue triangles)^1,26^, or ring-shaped guides (red circles)^27,28,33^. Green pentagons depict our twin-lattice interferometer in the current ① (*τ* = 12.1 ms, *K* = 408ℏ*k*) and a future ② version with improved interferometer parameters (*τ* = 48.4 ms, *K* = 808ℏ*k*).

Figure 4 shows the shape and size of areas employed in different gyroscopes as a function of their compactness factor. Typical butterfly geometries^1,26^ achieve high sensitivities but feature only little compactness. In our twin-lattice interferometer (*K* = 408ℏ*k*) with a total duration of *τ* = 2*T* = 12.1 ms, we enclose an area as large as *A* = 7.6 mm^2^ at a baseline of merely *L* = 2.43 mm. Overcoming current technical limitations, the realization of an interferometer with *τ* = 48.4 ms and a momentum separation of *K* = 808ℏ*k* at a contrast of *C* = 0.5 seems feasible. This would lead to an increased area of *A* = 240 mm^2^ and a baseline of *L* = 18.3 mm. In combination with an advanced atom source^41^ providing BECs of *N* = 10^5^ atoms at a 1 Hz rate, such a device would feature shot-noise limited sensitivities towards rotations and accelerations of 8 × 10^−9^ rad s^−1^ and 1.6 × 10^−9^ m s^−2^ per cycle. Hence, instead of requiring meters of baseline, this twin-lattice interferometer would fit into a volume <1 cm^3^. In terms of miniaturization it is comparable to guided structures^27,28,33^.

In conclusion, the symmetric nature and high scalability make twin-lattice atom interferometry a good candidate for applications requiring large space-time areas. Moreover, the demonstrated efficiencies recommend to combine twin-lattice interferometry with BECs featuring nonclassical correlations^42,43^. Besides gyroscopes^1,26^, our method is suitable for enhanced quantum tilt meters^18,44^, gradiometers^3^, and *h*/*m* measurements^4^. Last but not least, twin-lattice interferometry should open up the path to devices such as MIGA^6^ and ELGAR^8^ employing BECs with relative momenta of one thousand photon recoils as required for terrestrial detectors of infrasound gravitational waves^7^.

## Methods

### Twin-lattice laser system

For interferometry, we employ a frequency-doubled fiber laser system^45^ (NKT Photonics Koheras Boostik and Toptica Photonics SHG pro) 100 GHz blue-detuned from the ^87^Rb D_2_ line to reduce spontaneous emission. To create the twin lattice, we use two light fields with orthogonal linear polarizations, which are merged to a single beam on a polarizing beam splitter. Their frequencies and amplitudes are controlled independently by acousto-optical modulators (AOMs, AA Opto Electronics, MT80-A1.5-IR). The light is guided to the atoms via a single-mode fiber and collimated to a Gaussian-shaped beam of 3.75 mm waist and up to 1.2 W power (Thorlabs, F810APC-780). By retroreflecting the beam through a quarter-wave plate, the twin lattice is formed, that is, two lattices counterpropagating along the horizontal direction with orthogonal polarization to avoid standing waves. The lattices can be simultaneously accelerated in the opposite direction by chirping their frequency difference with the AOMs.

The radio-frequency signal for the AOMs is generated by two different devices. For BO, we employ a self-built device that is able to drive linear amplitude and frequency ramps at a sample rate of 250 kHz. For DBD, we use a pulse generator (PulseBlasterDDS-II-300-AWG) to form a Gaussian-shaped temporal intensity envelope.

### Bloch transfer efficiency in the twin lattice

**Fig. 5:**
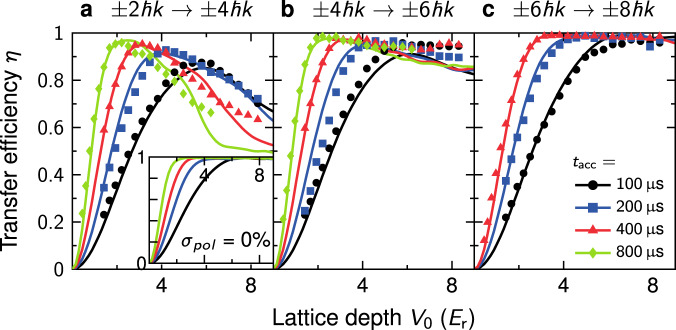
Bloch transfer efficiency in the twin lattice. Efficiency of one BO exerted by our twin lattice in dependence of the initial state. A superposition of BECs with momenta (**a**) ±2ℏ*k*, (**b**) ±4ℏ*k*, or (**c**) ±6ℏ*k* created via first-order DBD and sequential Bragg pulses is accelerated via BO acquiring an additional momentum of ±2ℏ*k*. The efficiency is recorded for different times *t*_acc_ and in dependence of the lattice depth *V*_0_ in units of recoil energy *E*_r_ = ℏ^2^*k*^2^/(2*m*). In all cases, a larger *t*_acc_ corresponds to a lower acceleration and reduces nonadiabatic losses as expected from Landau–Zener theory. Our experimental results (symbols) are well reproduced by theoretical simulations (lines) assuming an additional standing lattice of depth 0.37*V*_0_ arising from polarization imperfections (Supplementary Eq. (2)). The losses caused by the latter decrease with a larger initial splitting *K* and from (**a**) to (**c**) as the detuning from the standing lattice increases. The inset shows the theoretical efficiency for ideal polarization, that is, a perfect twin lattice, of the transfer ±2ℏ*k* → ±4ℏ*k*.

High efficiencies in the transfer of photon recoils are crucial for generating symmetric interferometers employing large momentum separation. Since, in case of a twin lattice, one lattice might disturb the effect of the other, we compare the efficiency of BO for different initial momentum separations of the BEC. Ideally, they should be equal to the ones of the individual lattices. Figure 5 illustrates nonadiabatic losses during BO in a twin lattice. We first prepare a superposition of symmetric momentum states ±2ℏ*k* (a), ±4ℏ*k* (b), or ±6ℏ*k* (c) by performing first-order DBD and a respective number of sequential first-order Bragg pulses. Subsequently, an additional momentum of ±2ℏ*k* is imparted via BO and the number of atoms in the final momentum states is measured. In particular, we compare the experimentally achieved (symbols) and the matching theoretically simulated efficiency (lines) for different durations *t*_acc_ = (100, 200, 400, 800) μs implying different accelerations in dependence of the lattice depth *V*_0_. In general, the transfer efficiency decreases for an increasing acceleration. Indeed, slower accelerations lower the rate of nonadiabatic interband transitions, and, hence, reduce atom losses in the final momentum state as expected from Landau–Zener theory^31^.

Figure 5a–c clearly shows the importance of the initial momentum separation prepared by DBD for an efficient transfer by the subsequent BO in our setup. While the efficiency for larger initial separations equals almost 100%, it is lower for smaller separations and also exhibits a pronounced optimum.

To explain the observed atom losses associated with the initial state, our simulations, based on a 1D-reduced Gross–Pitaevskii model^46^, had to take into account imperfections of our optical setup creating the twin lattice (Supplementary section 1). Neither the Landau–Zener theory describing an ideal single lattice nor our simulations of an ideal twin lattice, as shown in the inset (Fig. 5a) for the process ±2ℏ*k* → ±4ℏ*k*, could reproduce the experimental data. Indeed, these losses result from an imperfect polarization of the light fields giving rise to unwanted standing waves. Adding a standing lattice of 0.37*V*_0_ depth, which is compatible with our experimental setup, the theoretical curves agree with the experimental data. Fortunately, the losses can be overcome by increasing the initial momentum splitting of the BEC in our setup, which corresponds to a larger detuning to standing waves. In consequence, we start our interferometers with a beam splitter creating a superposition of ±4ℏ*k* (as in Fig. 5b), being the best trade-off between the losses caused by parasitic standing waves and the lower efficiency of Bragg processes.

Figure 2 depicts the temporal sequence regarding laser power and relative momentum for the combination of DBD with BOs in our twin lattice. For acceleration and deceleration of the BECs via BOs, the frequency difference of the twin lattice is linearly ramped up or down, respectively. For relative momenta of *K* = (24, 48, 88)ℏ*k* a single linear frequency ramp of 2 ms is employed. If the momentum transfer *K* in the interferometer is larger, the frequency ramp is split into two parts. For acceleration (deceleration) to relative momenta of *K* = (128, 208, 308, 408)ℏ*k*, a momentum of 12ℏ*k* is transferred during the first (final) (0.5, 0.4, 0.3, 0.3) ms and the rest in the remaining (1.5, 1.6, 1.7, 1.7) ms.

### Evaluation of spatial coherence

We analyze the spatial coherence of our twin-lattice interferometers statistically by measuring the interference amplitude in dependence of a spatial separation of the wave packet trajectories at the moment of the last DBD pulse^17^. The displacement between the wave packets is modified by varying the free-evolution time in the second half of the interferometer by ±*δT* as indicated in Fig. 2. The statistical analysis allows evaluating the spatial coherence in the presence of large vibrational noise. In our setup seismic measurements reveal noise as large as 10^−2 ^m s^−2^ Hz^−1/2^. Even in the case of interferometers involving momentum states as low as ±4ℏ*k* and a duration as short as 2*T* = 12.1 ms, this leads to a phase noise significantly larger than 2*π*. Hence, we assume that the population of the interferometer ports results from a random phase, that is, white phase noise.

Ideally, the displacement of the trajectories at the final DBD pulse is proportional to the time difference *δT* and the relative velocity *K*/*m*_Rb_. The dependence of the contrast on the displacement is described by a Gaussian bell-shaped curve $2\sqrt{2}{\sigma }_{p}(\delta T)\exp (-{(K/\hslash )}^{2}{\sigma }_{v}^{2}\delta {T}^{2}/2)$^17^, *σ*_*p*_ being the standard deviation of the normalized population *p* and *σ*_*v*_ the expansion rate of the BEC along the lattice. To calculate *σ*_*p*_ we take 40 data points for each *δT* (Supplementary Fig. 3).

**Fig. 6:**
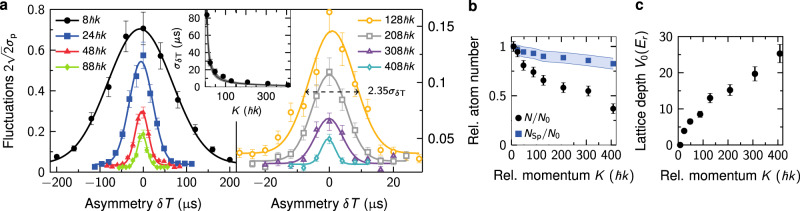
Interferometer analysis. **a** Measured atom number fluctuations $2\sqrt{2}{\sigma }_{p}$ of the twin-lattice interferometer in dependence of the spatial overlap of the wave packets at the last DBD pulse for increasing relative momenta *K* and corresponding Gaussian fits. The contrast is proportional to the standard deviation *σ*_*p*_ of the normalized atom number *p* at the output port. The error of *σ*_*p*_ is given by ${\sigma }_{p}(2(n-1))^{-1/2}$ with *n* = 40. We modify the spatial overlap by varying the duration of the second free-evolution time by *δT* relative to the first one. The displacement of the two wave packets varies with *δTK*. Inset: the width *σ*_*δT*_ of the envelopes decays with the inverse of the relative momentum *K* for a constant spatial coherence length. The line represents the theoretically calculated value *σ*_*δT*_ = ℏ/(*Kσ*_*v*_) for *σ*_*v*_ = (0.18 ± 0.03) mm s^−1^. **b** Relative atom number *N*/*N*_0_ measured in the output ports normalized to the DBD interferometer (*K* = 8ℏ*k*) (black circles) with an absolute error of 0.05. Also, calculated spontaneous emission decay *N*_Sp_/*N*_0_ (blue squares) with confidence intervals given by the errors in *N*/*N*_0_ and *V*_0_. **c** Lattice depth *V*_0_ applied during acceleration and deceleration with BOs with an assumed relative error of 0.1. Atom number and lattice depth serve as input parameters for our contrast simulation.

Figure 6a shows the experimentally determined value of *σ*_*p*_ depending on *δT* for different relative momenta *K* as well as the corresponding Gaussian fits to the data. The latter allows us to determine the maximum contrast *C* and the distribution’s width *σ*_*δT*_. For a given spatial coherence length, determined by *σ*_*v*_, the width *σ*_*δT*_ = ℏ/(*Kσ*_*v*_) ideally reduces with the inverse of the momentum separation *K* (inset in Fig. 6a). This agrees well with our observations and indicates that the coherent manipulation of the BEC is not reducing the coherence length of the atomic ensemble contributing to the interference signal.

### Input parameters for contrast simulation

In our case, the experimentally determined values of the lattice depth *V*_0_ during BO and the total atom number *N* detected in the output ports serve as input parameters for our simulation of the interference contrast in the twin lattice (Supplementary sections 3 and 4). We measure *N* relative to *N*_0_ ≡ *N*(8ℏ*k*) obtained in an interferometer exclusively based on DBD (Fig. 6b). The fraction *N*/*N*_0_ declines with relative momentum *K* to about 35% for *K* = 408ℏ*k* since nonadiabatic and spontaneous emission losses rise with increasing accelerations that require deeper lattices (Fig. 6c). We calculate *N*_Sp_, namely the atom number *N*_0_ diminished by spontaneous emission losses in a twin lattice of depth *V*_0_ (Supplementary section 2 for details). For example, in case of the *K* = 408ℏ*k* interferometer, the required lattice depth *V*_0_ = 25.3*E*_r_ leads to a spontaneous emission rate of 22 s^−1^ causing an atom loss of 1 − *N*_Sp_/*N*_0_ = 18%. In our model, spontaneous scattering equally affects atoms contributing to the signal and to the offset, and, hence, does not influence the contrast.

The confidence intervals result from a 10% error in *V*_0_ and an absolute error of 0.05 regarding *N*/*N*_0_.

## Supplementary information

Supplementary Information

## Data Availability

The data used in this manuscript are available from the corresponding authors upon reasonable request.

## Code availability

The simulation code is available from the corresponding authors upon reasonable request.
