# Supplementary material for: Twin-lattice atom interferometry
Source: Nat Commun. 2021 May 5;12:2544. doi: 10.1038/s41467-021-22823-8 (PMC8100166; doi:10.1038/s41467-021-22823-8)
Supplement: Supplementary file 1 — Supplementary Information [file 41467_2021_22823_MOESM1_ESM.pdf]

# Supplementary Materials for

## Twin-lattice atom interferometry

Martina Gebbe,[1,†,*] Jan-Niclas Siemß,[2,3,†,*] Matthias Gersemann,[2] Hauke Müntinga,[1,4]
Sven Herrmann,[1] Claus Lämmerzahl,[1] Holger Ahlers,[2,5] Naceur Gaaloul,[2]
Christian Schubert,[2,5] Klemens Hammerer,[3] Sven Abend,[2,*] Ernst M. Rasel[2]

[1]Zentrum für angewandte Raumfahrttechnologie und Mikrogravitation (ZARM),
Universität Bremen, Am Fallturm 2, D-28359 Bremen, Germany
[2]Institut für Quantenoptik, Leibniz Universität Hannover, Welfengarten 1, D-30167 Hannover, Germany
[3]Institut für Theoretische Physik, Leibniz Universität Hannover, Appelstr. 2, D-30167 Hannover, Germany
[4]German Aerospace Center (DLR), Institute for Satellite Geodesy and Inertial Sensing,
Am Fallturm 9, D-28359 Bremen, Germany
[5]German Aerospace Center (DLR), Institute for Satellite Geodesy and Inertial Sensing,
Callinstrae 36, D-30167 Hannover, Germany

[†]These authors contributed equally: Martina Gebbe and Jan-Niclas Siemß.
[*]Corresponding authors; E-mails: gebbe@zarm.uni-bremen.de,
jan-niclas.siemss@itp.uni-hannover.de, abend@iqo.uni-hannover.de.

**This PDF file includes:**

Section 1. Simulation of twin-lattice beam splitter efficiency.
Section 2. Spontaneous emission rate for atoms in moving optical lattices.
Section 3. Contrast reduction due to inefficiency of the momentum transfer.
Section 4. Contrast reduction due to distortions of the twin-lattice beam profile.
Fig. 1. Measured and simulated twin-lattice beam profile.
Fig. 2. Simulated contrast in dependence on the cloud size.
Fig. 3. Measured interferometer signal $p$ as a function of asymmetry $\delta T$.
Table 1. Comparison of areas enclosed by different Sagnac interferometers.

# Section 1. Simulation of twin-lattice beam splitter efficiency

The twin lattice is formed by the electric field $\mathbf{E}(y,t)$ propagating along the horizontal axis, here in $y$-direction. As depicted in Fig. 1 in the main text it comprises two frequencies $\omega_1$ and $\omega_2$

$$\mathbf{E}(y,t) = \left[E_1\boldsymbol{\epsilon}_1 \cos\left(k_1 y - \omega_1 t\right) + E_2\boldsymbol{\epsilon}_2 \cos\left(k_2 y - \omega_2 t\right)\right] \ , \tag{1}$$

and passes a quarter-wave plate before and after retroreflection. Ideally, the twin lattice features equal field amplitudes of both frequency components $E_0 \equiv E_1 = E_2$. We define $\Delta\omega = \omega_1 - \omega_2$ and note that $k \equiv k_1 \approx k_2$. Within the rotating wave approximation, the atom-light interaction gives rise to the spatially modulated dipole potential with depth $V_0$:

$$\begin{aligned} V(y,t) = V_0 \Bigg[ & \cos^2\left(ky - \Delta\omega t/2\right) + \cos^2\left(ky + \Delta\omega t/2\right) \\ & + \frac{\sigma_{\mathrm{pol}}}{(1-\sigma_{\mathrm{pol}})}\Big[2\cos\left(ky + \Delta\omega t/2\right)\cos\left(ky - \Delta\omega t/2\right)\Big]\Bigg] \end{aligned} \tag{2}$$

In addition to the twin lattice (given by the terms $\cos^2\left(ky \pm \Delta\omega t/2\right)$) Eq. (2) features an interference term resulting from polarization imperfections, acting as a standing lattice ($\propto \cos^2(ky)$) in terms of momentum transfer. Its magnitude depends on the scalar product of the polarization vectors $\boldsymbol{\epsilon}_1$ and $\boldsymbol{\epsilon}_2$: $\sigma_{\mathrm{pol}} \equiv \left|\boldsymbol{\epsilon}_1 \cdot \boldsymbol{\epsilon}_2\right|/2 \leq 0.5$.

We adapt a time-dependent Gross-Pitaevskii (GP) model [1] using the optical potential in Eq. (2) to calculate the efficiency of the momentum transfer depicted in Fig. 5 in the main text. The initial atomic state for our simulations is obtained by calibrating 3D numerical GP simulations to the experimentally observed free evolution of the atomic wave packet including the release from the magnetic trap and delta-kick collimation. In our simulations, double Bragg diffraction (DBD) realizing the initial relative momentum $K_{\mathrm{DBD}}$ is described by creating an ideal superposition of counter propagating wave packets in position space. The atomic state is multiplied with the phase factors $\frac{1}{\sqrt{2}}e^{\pm i\varphi_{\mathrm{DBD}}}$ (including normalization) providing the constituents

of the superposition, where $\varphi_{\rm DBD} = K_{\rm DBD}\, y/2\hbar$. Since our focus here is on the Bloch oscillation efficiency, we idealize the double Bragg interaction neglecting effects such as velocity selectivity or off-resonant couplings [2].

By changing the relative orientation of the polarization vectors we vary the strength of the undesired contributions to the dipole potential according to Eq. (2) which vanish in case of orthogonality. The theoretical curves presented in Fig. 5 in the main text have been obtained with $\sigma_{\rm pol} = 0.2688$ for all data sets corresponding to a standing wave depth of $0.37\ V_0$ (cf. second line in Eq. 2) which is a plausible assumption for the experimental setup. Polarization measurements have shown that the optical components, in particular the vacuum windows, degrade the polarization quality resulting in an extinction ratio of less than 20dB and a mismatch of several degrees from perfect orthogonality. The experimental values for $V_0$ in Fig. 5 in the main text have been calibrated via the Landau-Zener formalism.

# Section 2. Spontaneous emission rate for atoms in moving optical lattices

The spontaneous emission rate of an atomic transition of frequency $\omega_A$ interacting with the twin-lattice potential in Eq. (2) can in general be expressed as [3]

$$P = \frac{\Gamma}{|\Delta|}\,\frac{\langle V(y,t)\rangle}{\hbar}\ , \tag{3}$$

where $\Gamma$ is the natural linewidth of the transition and $\Delta \equiv \omega_{\rm L} - \omega_{\rm A}$ the detuning of the laser frequency $\omega_{\rm L}$ from resonance. The light creating the twin lattice is blue detuned ($\Delta > 0$) and we evaluate the spontaneous emission rate in the rest frame of the atomic wave packets. We do so by substituting $y \to y + \omega_{\rm D} t/k$ in $V(y,t)$, where $\omega_D = k\, v_{\rm BEC}$ depends on the atomic mean velocity $v_{\rm BEC}$.

Without loss of generality, we consider wave packets copropagating with the lattice traveling in

negative $y$-direction, i.e. $\omega_D = +\Delta\omega/2$, giving us the potential in the wave packet frame:

$$V(y,t) = V_0 \left[\cos^2(ky) + \cos^2(ky + \Delta\omega t) + \frac{\sigma_{\mathrm{pol}}}{(1-\sigma_{\mathrm{pol}})}\left[2\cos(ky+\Delta\omega t)\cos(ky)\right]\right] \quad (4)$$

In order to calculate the average spontaneous emission rate $P$ we consider the mean intensity an atom experiences during interaction with the lattice. We evaluate the contribution of the first term in Eq. (4) associated with the copropagating lattice by considering that the atomic wave function is largely overlapping with the nodes of the repulsive lattice potential. As detailed in [3], one can assume the atom to be well described by the lowest Bloch state of the comoving lattice to evaluate the average potential $\langle\cos^2(ky)\rangle = \frac{1}{2}\sqrt{E_{\mathrm{r}}/V_0}$, where $E_{\mathrm{r}} = \hbar^2k^2/(2m)$ is the recoil energy. Given that the frequency within the cosine arguments $\Delta\omega$ is typically much greater than the rate $\frac{\Gamma}{|\Delta|}\frac{V_0}{\hbar}$ in Eq. (3) we can take the temporal average of the other terms $\langle\cos^2(ky+\Delta\omega t)\rangle \approx \frac{1}{2}$ and $\langle\cos(ky+\Delta\omega t)\cos(ky)\rangle \approx 0$, respectively. Inserting these results into Eq. (3) leads us to the total emission rate for an atom that copropagates with one of the twins

$$P = \frac{\Gamma}{|\Delta|}\frac{V_0}{\hbar}\left[\frac{1}{2\sqrt{V_0/E_{\mathrm{r}}}} + \frac{1}{2}\right] . \quad (5)$$

After a total duration $\tau_{\mathrm{twin}}$ of the interaction with the twin lattice, the atom number $N_{\mathrm{Sp}}$ thus decreases with rate $P$ as

$$N_{\mathrm{Sp}} = N_0 \cdot e^{-P\cdot\tau_{\mathrm{twin}}} , \quad (6)$$

where $N_0$ is the atom number measured in the output ports of the interferometer solely based on double Bragg diffraction ($K = 8\hbar k$). Figure 6b in the main text displays the ratio $N_{\mathrm{Sp}}/N_0$ in dependence of the relative momentum $K$. As $\tau_{\mathrm{twin}}$ is identical for all our interferometer sequences featuring Bloch oscillations, the spontaneous emission rate $P$ only depends on the lattice depth $V_0$ (Fig. 6c in the main text).

# Section 3. Contrast reduction due to inefficiency of the momentum transfer

Atom losses during Bragg diffraction and Bloch oscillations degrade the contrast by reducing the coherent fraction of atoms which contribute to the interferometer signal. To estimate the contrast loss, we make the following assumptions to simplify our calculations: (i) A finite double Bragg beam splitting fidelity leads to an offset $N_{\mathrm{OS}}$ in the output ports and, thus, to a reduction of the interference amplitude. (ii) Spontaneous scattering equally affects the coherent fraction of atoms as well as the offset in the interferometer ports and, hence, does not lead to a contrast decay in our case. Since we are using delta-kick collimated BECs with a momentum width far below the photon recoil, the vast majority of spontaneously scattered atoms is separated from the output ports, anyway, and not counted by our spatial detection system. (iii) Landau-Zener losses during Bloch oscillations remove atoms from the interferometer and, therefore, lead to loss of contrast.

The contrast can be expressed by the maximum $p_{\max}$ and minimum $p_{\min}$ normalized number of atoms detected in the interferometer ports:

$$C = 2\sqrt{2}\sigma_p \approx \frac{p_{\max} - p_{\min}}{p_{\max} + p_{\min}}, \tag{7}$$

where $\sigma_p$ is the standard deviation of the normalized population $p$ defined in the main text. We attribute the contrast of our double Bragg interferometer, $C(K = 8\hbar k) = 0.7059 \pm 0.022$, solely to the finite efficiency of the double Bragg beam splitting processes. Due to a similar fidelity for first and sequential Bragg diffraction the offset $N_{\mathrm{OS}}$ is assumed to be equally distributed between the inner ($N_{0\hbar k}$) and the outer ports ($N_{\pm 2\hbar k}$). We write the atom number in the output ports $N$ as the sum of the signal $N_{\mathrm{Sig}}$ and the offset $N_{\mathrm{OS}}$, $N(K) = N_{\mathrm{Sig}}(K) + N_{\mathrm{OS}}(K)$.

Our contrast model hinges on the following assumptions: (i) The double Bragg processes are elements of all our interferometers and, hence, $N_{\mathrm{OS}}$ is identical for all $K$. The extreme

values for $p$ thus equal

$$p_{\min} = \frac{N_{\mathrm{OS}}(K)/2}{N(K)} \ ,$$
$$p_{\max} = \frac{N_{\mathrm{Sig}}(K) + N_{\mathrm{OS}}(K)/2}{N(K)} \ .$$

Inserting these into Eq. (7) leads to

$$C(K) = \frac{N_{\mathrm{Sig}}(K)}{N_{\mathrm{OS}}(K) + N_{\mathrm{Sig}}(K)} \ . \tag{8}$$

Since we assume an equal spontaneous emission rate for all momentum classes, $N_{\mathrm{OS}}$ decreases with rate $P$ in the presence of Bloch oscillations and can be written as

$$N_{\mathrm{OS}}(K) = N_{\mathrm{OS}}(8\hbar k) \cdot e^{-P(K)\cdot\tau_{\mathrm{twin}}} = (1 - C(8\hbar k))N_0 \cdot e^{-P(K)\cdot\tau_{\mathrm{twin}}} = (1 - C(8\hbar k))N_{\mathrm{Sp}}(K) \ .$$

(ii) Acceleration of the atoms with Bloch oscillations lowers the signal $N_{\mathrm{Sig}}(K)$ due to non-adiabatic transitions. The offset $N_{\mathrm{OS}}$ is only affected by spontaneous emission, but not by non-adiabatic losses, since offset atoms do not perform Bloch oscillations and Landau-Zener losses are expected to be spatially well separated from the output ports. A decrease in $N_{\mathrm{Sig}}(K)$ therefore reduces the contrast according to Eq. (8).

Combining these assumptions we express the contrast in Eq. (8) as a function of $K$ that requires as input parameters the experimentally determined ratio $N(K)/N_{\mathrm{Sp}}(K)$ (Fig. 6b in the main text) as well as the measured contrast $C(8\hbar k)$:

$$C(K) = \frac{N(K) - N_{\mathrm{OS}}(K)}{N(K)} = 1 - \frac{1 - C(8\hbar k)}{N(K)/N_{\mathrm{Sp}}(K)} \ . \tag{9}$$

The green diamonds in Fig. 3 in the main text depict the results of Eq. (9).

# Section 4. Contrast reduction due to distortions of the twin-lattice beam

We study the reduction of the interference contrast due to spatial intensity fluctuations of the twin-lattice laser beam (Supplementary Fig. 1) deforming the atomic trajectories and causing

imperfect mode overlap either in momentum or in position space at $t = 2T$.

Experimental observations suggest, that a non-ideal spatial overlap in the direction of the twin lattice can be excluded as it would introduce a timing asymmetry noticeable in the contrast envelopes in Fig. 6a in the main text.

We investigate the impact of unequal momenta in both interferometer arms, e.g. $\Delta p_y$ in $y$-direction, which leads to a spatially dependent phase difference $\delta\varphi_y = \Delta p_y \cdot y/\hbar$, and local interference fringes with spacing $2\pi\hbar/\Delta p_y$ in the output ports upon spatial imaging. For fringe spacings significantly smaller than the size of the atomic clouds this unwanted phase contribution reduces the measured contrast.

In our model, a path-dependent dipole force acts on the atoms via the gradient of the distorted lattice beam potential

$$U(x, y, z) = U_0(K) I(x, y, z)/I_0, \tag{10}$$

where $I(x, y, z)/I_0$ is the normalized intensity distribution of the Gaussian lattice beam that has been diffracted at the edge of the atom chip [4] (Supplementary Fig. 1c) and $U_0(K) = V_0(K)/(1 - \sigma_{\mathrm{pol}})$ is the total potential depth of the beam. Integration of this dipole force for the interferometer duration $2T$ along each arm reveals a differential momentum between the two arms $\Delta p_j = p_{j,\mathrm{arm1}} - p_{j,\mathrm{arm2}}$ with $j = x, y, z$

$$\begin{aligned} p_{j,\mathrm{arm}}(K) &= -\int_0^{2T} \frac{\partial}{\partial j} U(x_{\mathrm{arm}}(t, K), y_{\mathrm{arm}}(t, K), z_{\mathrm{arm}}(t, K))\, \mathrm{d}t \\ &= -\frac{V_0(K)}{I_0} \int_0^{2T} \frac{\partial}{\partial j} I(x_{\mathrm{arm}}(t, K), y_{\mathrm{arm}}(t, K), z_{\mathrm{arm}}(t, K))\, \mathrm{d}t\ . \end{aligned} \tag{11}$$

$p_{j,\mathrm{arm}}$ depends on the momentum separation $K$ via the wave packet trajectories (Fig. 2 in the main text) and the lattice depth $V_0$ (Fig. 6c in the main text). We evaluate Eq. (11) for the intensity distribution of the distorted Gaussian beam making the assumption that the atomic motion during the twin-lattice interferometer is given by simplified linearly accelerated trajectories.

The interferometric contrast $C_{\mathrm{LD}}$ for a particular value of $\Delta p_j(K)$ is calculated with the following integral:

$$C_{\mathrm{LD}}(K) = \left| \iiint \left| \Psi_{(0\hbar k, \pm 2\hbar k)}(x, y, z, t = 2T) \right|^2 \; e^{-\frac{i}{\hbar}(\Delta p_x \cdot x + \Delta p_y \cdot y + \Delta p_z \cdot z)} \mathrm{d}x \mathrm{d}y \mathrm{d}z \right|. \qquad (12)$$

Since the twin-lattice laser beam is well collimated and there is very little atomic motion in the $x$-direction compared to both the $y$- and $z$-directions, we assume the beam profile to be symmetric in $x$-direction simplifying our calculations by setting $\Delta p_x = 0$.

We model the density $\left|\Psi_{(0\hbar k, \pm 2\hbar k)}(x, y, z, t = 2T)\right|^2$ of the two interfering wave packets at the interferometer output ports with momenta $0\hbar k$ and $\pm 2\hbar k$ by using Thomas-Fermi density distributions. Their sizes have been inferred from time-of-flight measurements. In order to account for a dependence of $\Delta p_j(K)$ on the spatial extent of the atomic wave packet we calculate a sample of single-particle trajectories with different initial positions given by density distribution at the beginning of the interferometer sequence $|\Psi_{\mathrm{I}}(x, y, z, t = 0)|^2$ and average the contrast values in Eq. (12) over those realizations providing an average light-diffraction contrast $C_{\mathrm{LD,avg}}(K)$. The loss of the contrast due to path-dependent dipole forces can be seen as the consequence of increasingly random velocity variations [5]. For our simulated light field we obtain average differential velocities $\delta v$ on the order of tens of nm/s (a few $\mu$m/s) in the y-direction (in z-direction) amounting to negligible phase shifts. The standard deviation of $\delta v$, however, ranges from tens of m/s for $K = 24\,\hbar k$ up to several hundreds of $\mu\mathrm{m/s}$ for $K = 408\,\hbar k$. This results in uncertainties in the phase ranging from a few hundred mrad ($K = 24\,\hbar k$) up to 2 rad ($K = 408\,\hbar k$) over the cloud at which point the contrast is almost diminished.

To match our simplified model with the experiment, we use a single fitting parameter, namely a factor multiplying the normalized intensity ratio in Eqs. (10) and (11). Our simplified light distortion model features intensity perturbations $|I(x, y, z) - I_{\mathrm{Gauss}}|/I_0$ of about $1\%$ caused by the diffraction on the metallic edge (Supplementary Fig. 1c). The contrast values

(red triangles) depicted in Fig. 3 in the main text have been obtained enhancing the perturbations to 9% providing good agreement with the experiment, where intensity fluctuations in the order of 10% have been measured.

Regarding our model one has to note, that assuming a beam clipped on a single chip edge oversimplifies the experimental situation, where diffraction occurs at different apertures causing larger total distortions. A 3D simulation of the totality of these diffractions requires a more detailed study and goes beyond the scope of this work. Direct measurements of the beam profile outside of the vacuum chamber as depicted in Supplementary Fig. 1a, however, show intensity variations at the level of 10% and, thus, support our assumptions. In turn, increasing the intensity of an ideal Gaussian beam does not lead to a significant loss of signal in our model. To maintain a contrast of $C_{\mathrm{LD,avg}} = 90\%$ up to $K = 408\hbar k$ the intensity fluctuations should not exceed 0.5%. The confidence intervals in Fig. 3 in the main text reflect the 10% uncertainty in the measured lattice depth $V_0(K)$. The orange pentagons represent the product of the results of Eqs. (9) and (12), $C_{\mathrm{total}}(K) = C_{\mathrm{LD,avg}}(K) \cdot C(K)$.

| | Type | $A$ (mm$^2$) | $\tau$ (ms) | $L$ (mm) | Calculation of $A$ and $L$ |
|---|---|---|---|---|---|
| Twin lattice | | 7.6 | 12.1 | 2.43 | $L = \frac{1}{2}g((\tau + t_0)^2 - t_0^2)$ |
| Savoie [6] | Butterfly | 1120 | 801 | 787 | $A = \frac{1}{2}v_{\mathrm{r,Cs}}g(\tau/2)^3$, $L = \frac{1}{2}g(\tau/2)^2$ |
| Stockton [7] | Butterfly | 19 | 206 | 52 | $A = \frac{1}{2}v_{\mathrm{r,Cs}}g(\tau/2)^3$, $L = \frac{1}{2}g(\tau/2)^2$ |
| Berg [8] | Mach-Zehnder | 41 | 50 | 140 | $A = \tau^2 v v_{\mathrm{r,Rb}}$, $L = \tau v$ |
| Canuel [9] | Mach-Zehnder | 2.1 | 60 | 19.8 | $A = 2(\tau/2)^2 v v_{\mathrm{r,Cs}}$, $L = \tau v$ |
| Moan. [10] | Ring-shaped guide | 0.5 | 107.7 | 0.4 | $A = 4\pi R^2$, $L = 2R$ |
| Pandey [11]* | Ring-shaped guide | 25.3 | 2000 | 0.886 | $A = 41 \cdot \pi R^2$, $L = 2R$ |
| Wu [12] | Moving guide | 0.18 | 50 | 1 | $A = L/\pi 2 v_{\mathrm{r}} \tau$ |

* No realization of an interferometer

Table 1: **Comparison of areas enclosed by different Sagnac interferometers**. Parameters used to calculate the area $A$ and compactness factor $(\tau L)^{-1}$ in Fig. 4 in the main text. Whenever possible, a formula is given for the calculation of the area $A$ and the baseline $L$. $t_0$ is the free-fall time before the interferometer, $v_{\mathrm{r,Rb/Cs}} = \hbar k/m_{\mathrm{Rb/Cs}}$ the recoil velocity and $v$ the forward velocity perpendicular to both gravity $g$ and the wave vector $k$.

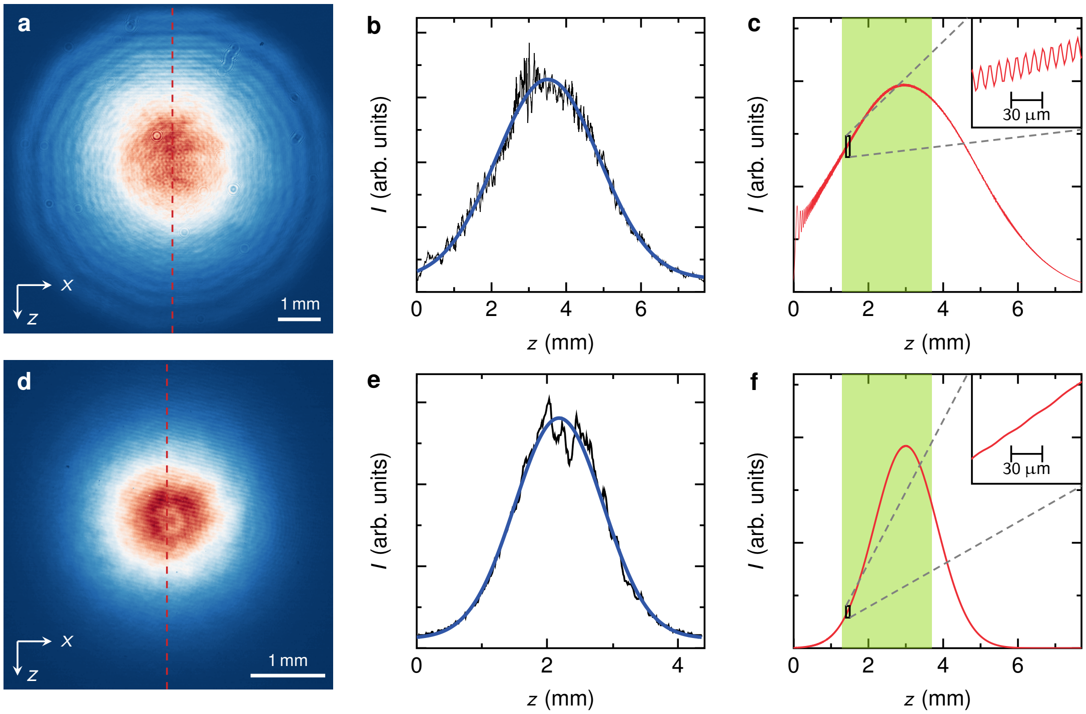


Fig. 1: **Measured and simulated twin-lattice beam profile.** (**a**) Twin-lattice beam with a waist of 3.75 mm imaged with a beam profiling camera after passing through the vacuum chamber. Interference fringes arise due to clipping at apertures and the atom chip. (**b**) Vertical intensity profile (black line) along the dashed red line indicated in (**a**) as well as Gaussian fit to the data (blue line). (**c**) Simulated intensity profile of a Gaussian beam with $w = 3.75$ mm diffracted at the edge of a metallic half-plane [4]. The interferometer region is shaded in green. The inset compares the scale of the intensity fluctuations to the cloud's Thomas-Fermi radius of roughly 30 $\mu$m. (d,e,f) Measured beam and beam profile of the smaller beam with $w = 1.65$ mm as well as simulated intensity profile analog to (a,b,c).

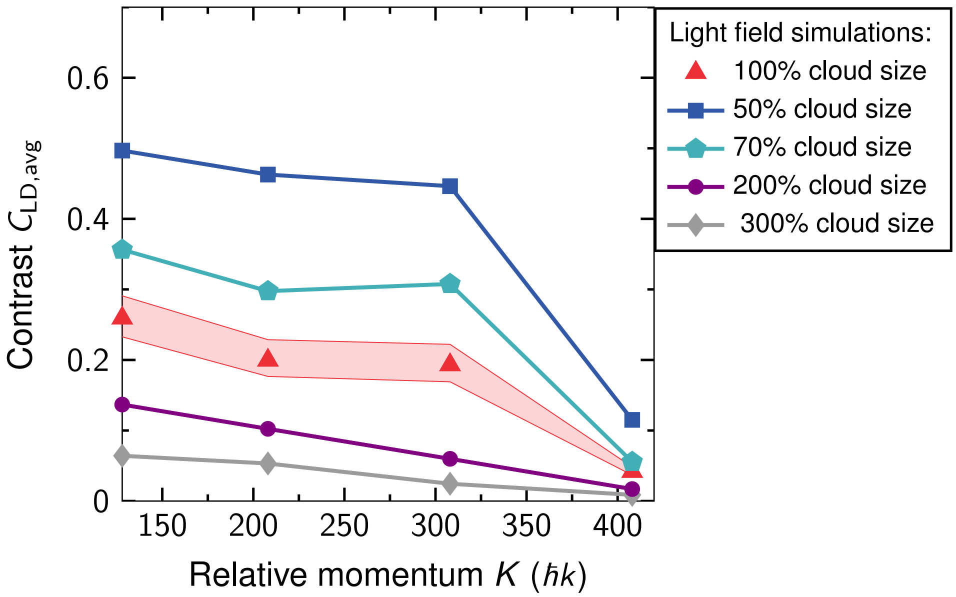


Fig. 2: **Simulated contrast $C_{LD,avg}(K)$ in dependence of cloud size.** We show the calculated contrast reduction due to the interaction of the BEC with the distorted twin-lattice beam according to Eq. (12) (red triangles in Fig. 3 in the main text) for large relative momenta $K > 100\hbar k$. We compare the contrast $C_{LD,avg}(K)$ of our delta-kicked collimated BEC (100% cloud size, red triangles) to values calculated for different cloud sizes but otherwise identical input parameters. A magnification of the cloud's spatial spread to 200% or 300% (violet circles, gray diamonds) increases the phase variation across the cloud and, therefore, leads to a significant contrast reduction. Clouds with smaller radii of $70\%$ (turquoise pentagons) and $50\%$ (blue squares) in turn provide improved contrast highlighting the benefit of a well-collimated atom source in the presence of light field distortions.

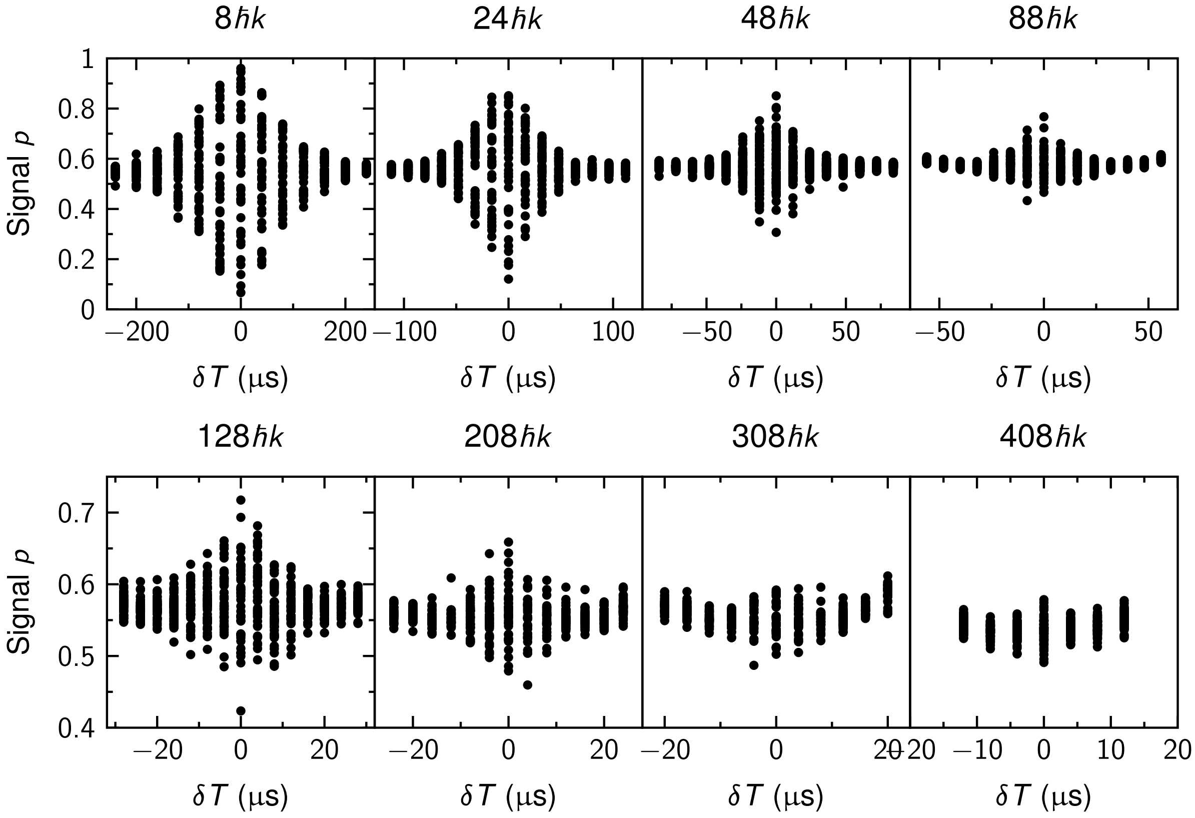


Fig. 3: **Measured interferometer signal $p$ as a function of asymmetry $\delta T$.** We plot the signal $p = (N_{+2\hbar k} + N_{-2\hbar k})/(N_{+2\hbar k} + N_{-2\hbar k} + N_{0\hbar k})$ depending on $\delta T$ for different relative momenta $K = (8, 24, 48, 88, 128, 208, 308, 408)\,\hbar k$ in the twin-lattice interferometer. For each $\delta T$ we take 40 data points to calculate the standard deviation $\sigma_p(\delta T)$ of $p$ (Fig. 6a in the main text).

# References


[1] Salasnich, L., Parola, A. & Reatto, L. Effective wave equations for the dynamics of cigar-shaped and disk-shaped Bose condensates. *Phys. Rev. A* **65**, 043614 (2002).

[2] Hartmann, S. *et al.* Regimes of atomic diffraction: Raman versus Bragg diffraction in retroreflective geometries. *Phys. Rev. A* **101**, 053610 (2020).

[3] Cladé, P. *et al.* Precise measurement of $h/m_{\mathrm{Rb}}$ using Bloch oscillations in a vertical optical lattice: Determination of the fine-structure constant. *Phys. Rev. A* **74**, 052109 (2006).

[4] Anokhov, S. P., Lymarenko, R. A. & Khizhnyak, A. I. Wide-angle diffraction of the laser beam by a sharp edge. *Radiophysics and Quantum Electronics* **47**, 926–932 (2004).

[5] Charrière, R., Cadoret, M., Zahzam, N., Bidel, Y. & Bresson, A. Local gravity measurement with the combination of atom interferometry and Bloch oscillations. *Phys. Rev. A* **85**, 013639 (2012).

[6] Savoie, D. *et al.* Interleaved atom interferometry for high-sensitivity inertial measurements. *Science Advances* **4**, eaau7948 (2018).

[7] Stockton, J., Takase, K. & Kasevich, M. A. Absolute geodetic rotation measurement using atom interferometry. *Phys. Rev. Lett.* **107**, 133001 (2011).

[8] Berg, P. *et al.* Composite-light-pulse technique for high-precision atom interferometry. *Phys. Rev. Lett.* **114**, 063002 (2015).

[9] Canuel, B. *et al.* Six-axis inertial sensor using cold-atom interferometry. *Phys. Rev. Lett.* **97**, 010402 (2006).

[10] Moan, E. R. *et al.* Quantum rotation sensing with dual Sagnac interferometers in an atom-optical waveguide. *Phys. Rev. Lett.* **124**, 120403 (2020).

[11] Pandey, S. *et al.* Hypersonic Bose-Einstein condensates in accelerator rings. *Nature* **570**, 205–209 (2019).

[12] Wu, S., Su, E. & Prentiss, M. Demonstration of an area-enclosing guided-atom interferometer for rotation sensing. *Phys. Rev. Lett.* **99**, 173201 (2007).
